# Genome Paths: A Way to Personalized and Predictive Medicine

**Published:** 2009-10

**Authors:** V. S. Baranov

**Affiliations:** 1 Ott's Institute of Obstetrics and Gynecology, Russian Academy of Medical Sciences

## Abstract

The review is devoted to the impact of human genome research on progress in modern medicine. Basic achievements in genome research have resulted in the deciphering of the human genome and creation of a molecular landmarks map of the human haploid genome (HapMap Project), which has made a tremendous contribution to our understanding of common genetic and multifactorial (complex) disorders. Current genome studies mainly focus on genetic testing and gene association studies of multifactorial (complex) diseases, with the purpose of their efficient diagnostics and prevention . Identification of candidate ("predisposition") genes participating in the functional genetic modules underlying each common disorder and the use of this genetic background to elaborate sophisticated measures to efficiently prevent them constitutes a major goal in personalized molecular medicine. The concept of a genetic pass as an individual DNA databank reflecting inherited human predisposition to different complex and monogenic disorders, with special emphasis on its present state, and the numerous difficulties related to the practical implementation of personalized medicine are outlined. The problems related to the uncertainness of the results of genetic testing could be overcome at least partly by means of new technological achievements in genome research methods, such as genome-wide association studies (GWAS), massive parallel DNA sequencing, and genetic and epigenetic profiling. The basic tasks of genomic today could be determined as the need to properly estimate the clinical value of genetic testing and its applicability in clinical practice. Feasible ways towards the gradual implementation of personal genetic data, in line with routine laboratory tests, for the benefit of clinical practice are discussed.

## INTRODUCTION

The revolutionary achievements in human genetics such as full genome seguencing, the successful completion of the HapMap program (haploid genome), the rapid development of bioinformatics and nanotechnology, advances in the delelopment of efficient methods for genome analysis are signs of a new era: the era of genomics. The 21^st^ century could well be remembered as the century of genetics [1, 2]. Impressive progress in comparative and functional genomics has assured the widespread introduction of this branch in medicine. Thus, it has led to the emergence and rapid development of medical genomics in which such problems of classical medicine as diagnosis, prevention, and treatment are solved at the level of nucleic acids and the products of their expression: RNA and proteins [3, 4, 5]. The preventive direction in molecular medicine gave rise to predictive medicine (PM). Its main features appear to be its individual (the genome of every human individual) and preventive character (analyses of the genome are possible at any stage of ontogeny, long before the onset of a particular disease). The main principles of predictive medicine and genetic testing (GT) as a methodological basis for PM, as well as the concept of a "genetic passport," were formulated by us in 2000 [5, 6, 37].

## 1. POLYMORPHISM OF GENES AS THE BASIS OF PREDICTIVE MEDICINE

The genomes of all people, except for identical twins, are different. Pronounced population, ethnic, and, most importantly, individual genome features in translatable parts of genes (exons), as well as in their noncoding sequences (intergenic spaces and introns), are caused by mutations leading to genetic polymorphism (GP). The latter is usually defined as a mendelian trait occurring in the population, at least in two variants, with a frequency of not less than 1% for each one [10]. GPs may be quantitative or qualitative.

Quantitative GP is represented by facultative elements, which account for up to 50% of the entire genome. These are micro- and minisatellite DNA, as well as DNA composed of tandem repeats (STR - Short Tandem Repeats), retrotransposons, and extensive repeats with variability in core nucleotide sequence composition - VNTR (Variable Number Tandem Repeats). Finally, in recent years, thanks to new methods of DNA analysis (CNV - Copy Number Variation and GWAS - Genome Wide Association Studies), the existence of polymorphism in large DNA fragments (1-50 MGB) was demonstrated in the human genome; it is the so-called Copy Number Variation - CNV.

Qualitative GP is mainly single nucleotide polymorphism (SNP). This most frequent GP occurs roughly every 300-400 bp. Thus, the total number of SNP in the full human genome is estimated at about 10-13 · 10^6^. There is a surprising similarity (99.9%) in GP in the genomes of different people. Stable combination of several neighboring alleles - SNP on one strand of DNA (haplotype) - has allowed to use them as specific molecular markers in the program HapMap (haploid map) (see below).

It is assumed that about half of all SNP (5 million) are related to the sense (expressed) part of the genome. It is these substitutions that correspond to the allelic variants of genes that cause or are associated with various diseases. They play a key role in human GP [5, 7, 8, 9].

It is well known today that polymorphism is typical for almost all human genes. It has been established that it has a clear ethnic and population specificity. Polymorphisms affecting the coding parts of genes often lead to the replacement of amino acids and the appearance of proteins with new functional properties. Replacements or nucleotide repeats in the regulatory (promoter) regions of genes may have a significant impact on gene expression. Inherited gene changes play a crucial role in determining the unique biochemical profile of each individual, his or her hereditary predisposition to various MD.

## 2. HAPLOID MAP (HAPMAP) PROGRAM

The key role in the study of GP is played by the international project for the study of the haploid human genome - Haploid Map (HapMap).

The purpose of the project is to obtain a genetic map of the distribution of single-nucleotide substitutions (SNP) in the haploid set of all 23 human chromosomes [11]]. The main thrust of the project consists in that the analysis of the distribution of known SNP in individuals for several generations revealed that SNPs that are adjacent or closely located in the DNA of one chromosome are inherited as SNP blocks. This block represents a SNP haplotype - a set of alleles located on the same chromosome (hence the origin of the name of the project HapMap). Thus, each of the mapped SNP acts as an independent molecular marker. By linking these SNP-markers with the manifestations being investigated (disease symptoms), the most likely location of candidate genes’ mutations (polymorphisms) associated with a particular MD are identified. Typically, there are 5 or 6 SNPs closely linked with the already known Mendelian traits selected for mapping. Well described SNPs with a frequency of rare alleles of not less than 5% are called marker SNPs (tagSNP). It is assumed that, ultimately, in the process of the project’s implementation only about 500 000 tagSNP of the approximately 10 million SNPs present in the genome of every individual will be selected. But this number is more than enough in order to map with SNPs the full human genome and identify new candidate genes linked with various MDs [12].

Thanks to HapMap, which includes the SNPs of not only already known genes, but also those of genes that have yet to be identified, scientists now have at their disposal the powerful universal navigator needed for the in-depth analysis of the genome of each individual for a fast and efficient mapping of genes whose allelic variants predispose one to various MDs (see below).

Francis Collins, the director of the National Institute for the Study of the Human Genome (USA) says: "Already 20 years ago when discussing the program ’Human Genome’, I dreamed of a time when the genomic approach will become a tool for the diagnosis, treatment and prevention of severe common diseases that affect patients overcrowding our hospitals and doctors’ offices. The success of the HapMap project allows us to make a significant step toward this dream already today."

## 3. GENES AND HUMAN DISEASES

According to research in the prevalence of diseases in twin pairs and medical genetics data, only about 1.5% of human diseases are directly linked to mutations. These are so-called hereditary diseases. Accuracy in the molecular diagnosis of hereditary diseases is very high and approaches 100%.

All other diseases, including such frequent ones as cardiovascular, cancer, mental, and even infectious diseases, are the result of the combined effect of unfavourable external factors and individual characteristics of the genome, somehow predisposing a particular person to a disease. Hence, the origin of their name: multifactorial (combined or complex) diseases (MD).

Thanks to full human genome seguencing, the development of convenient methods for the mapping of new genes (the total number of genes in humans is estimated at about 22,000), methods for identifying mutations, as well as the problem of diagnosing multiple (especially the most frequent) monogenic human genetic diseases (cystic fibrosis, hemophilia, Duchenne/Baker muscular dystrophy, spinal muscular atrophy, immunodeficiency and others) can be considered as solved [13, 14, 15]. The identification of the genes involved in the genesis of MDs, the so-called "predisposition genes," is much more complicated. According to the current definition, "predisposition genes" are mutant genes (alleles) that are compatible with birth and life but that, under certain unfavorable conditions, can contribute to the development of a MD [14].

**Fig. 1.**
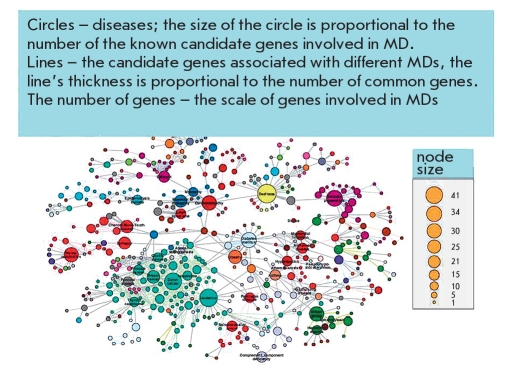
Gene networks of some common multifactorial (complex) disorders

**Fig. 2.**
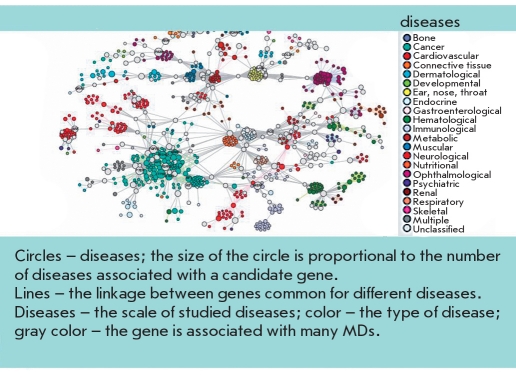
Functional genetic modules of different MDs

**Fig. 3.**
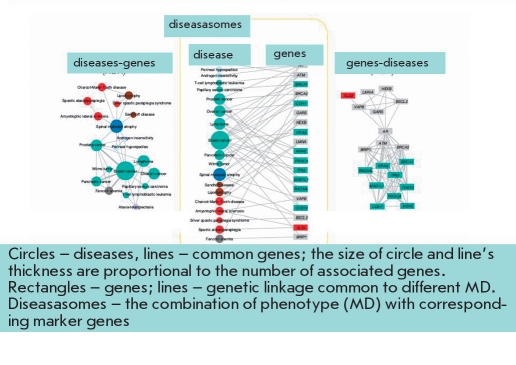
The topology of metabolic networks and the genetics of complex disorders

**Fig. 4.**
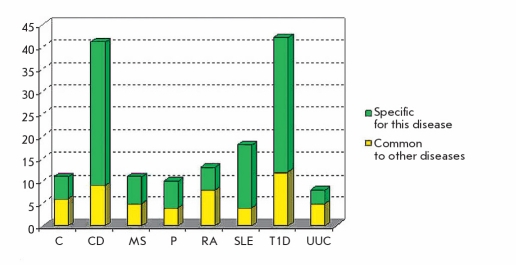
The number of loci common to autoimmune diseases, and loci that are specific to a particular disease Conventional signs: C - celiac disease, CD - Crohn’s disease, MS - multiple sclerosis, P - psoriasis, RA - rheumatoid arthritis, SLE - systemic lupus erythematosus, T1D - type 1 diabetes, UUC - unspecific ulcerative colitis

Depending on participation in metabolic chains and associations with a MD, susceptibility genes are conditionally divided into several groups, among which are the genes of the detoxification system ("external environment"), the genes of metabolic bypass (genes triggers), cell receptor genes, genes of the inflammation and immune system, and genes associated with a specific MD [Baranov, 2000, Baranov et al, 2000]. Unfavorable allelic variants of these genes can cause atherosclerosis, coronary heart disease (CHD), osteoporosis, diabetes, asthma, tumors, etc. The combinations of allelic variants of different genes that provide normal metabolic processes or are involved in the development of a specific multifactorial pathology are called "gene networks"[16]. In each of these networks, the main (central) genes and additional (auxiliary) genes (the so-called genes modifiers) are defined. The concept of genetic networks has further evolved to the study of functional genetic modules (FGM). To this end, a series of studies have compared the MDs and the various genes whose products are involved in the etiology and pathogenesis of these diseases [17, 18, 19]. A network of MDs and the genes common to these diseases was created and named the human disease network (HDN) (Fig. 1) and, vice versa, a network of genes common to various diseases - disease genetic network - DGN (Fig. 2) has also been created. Combining HDN-and DGN-maps has made it possible to create the so-called diseasasome map that reflects the topology of metabolic networks and the genetics of MDs (Fig. 3). As a result of a large-scale study of 1,264 MDs and their associated 1,777 genes [17], the following has been established: 1) each MD is characterized by its own set of genes - a gene network or so-called functional gene modules (FMG), there are central and peripheral genes that can be distinguished by module; 2) most MDs are interconnected through many different genes; 3) 516 MDs show a lot of genetic linkages; ie, they are associated with many genes (deaf - 41 genes, leukemia - 37, cancer of the colon - 34); 4) the mutation of different genes can lead to the same MD, and mutations (polymorphisms) of one gene may be associated with different MDs; 5) the mutation of the central (essential) genes of FGM are often associated with tumors and causes early death; 6) mutations (polymorphisms) of the peripheral FMG genes play a major role in the phenotypic variability and the development of MDs; 7) the presence of overlapping FGM of MDs shows the pathogenetic proximity of different MDs and argues in favor of syntropy - a combination of pathogenetically related, "family," of MDs; and 8) genes included in FGM are essentially "syntropy" genes [20] which are functionally similar but not always identical to "predisposition" genes. The coincidence of many MDs in a large number of associated genes was clearly demonstrated when comparing candidate genes associated with various autoimmune diseases (Fig. 4).

About a third of the identified loci are associated with two, three, or more diseases. The presence of more than 5% of common candidate genes associated with the celiac disease, Crohn’s disease, multiple sclerosis, psoriasis, rheumatoid arthritis, systemic lupus erythematosus, diabetes mellitus type-1, and ulcerative colitis proves the pathogenetic similarity of these autoimmune diseases [21, 22] and allows one to consider them as a single group of sintropy diseases.

Clarification of th eFGM of each MD, identification of the central genes and modifier genes in it, the analysis of the association of the alleles of these genes with the disease, development of a set of preventive measures for persons at high risk and, subsequently, for a particular patient on this basis is the main focus of predictive medicine [5].

## 4. SEARCH STRATEGY FOR "PREDISPOSITION" GENES

In past decades the search for candidate genes has been performed by means of two approaches: the analysis of association and analysis of linkage.

The basis of the method of analysis of the association is the disequilibrium in the linkage between the mutation and the nearby marker tested [23]. The method involves several steps: 1) selecting the most probable candidate genes on the basis of the particular disease, 2) the selection of functionally important alleles of the corresponding genes, 3) a population analysis of the allele and genotype frequencies of the corresponding genes on the basis of literature data and the Internet, and 4) a comparative analysis of allele frequencies and genotypes of these genes in patients with a clinically confirmed diagnosis and in healthy individuals of the same population, matched by the type of "experience-control."

Studies are conducted on representative samples of patients and donors (at least 100 people in each group).

Obviously, such a way, being quite lengthy, laborious and costly, at the same time does not guarantee that the identified allelic differences are the main ones in the chain pathogenetic mechanisms involved in the particular disease. It does not rule out the fact that some important genes and polymorphisms of the same, or even more, or another gene network involved in the disease can be missed, and that clinically different forms of the disease under study may have a different pattern of candidate genes.

An alternative strategy of searching for predisposition genes (linkage analysis) is based solely on the positional cloning of locus and does not require a preliminary hypothesis about the pathophysiology of the disease. Initially, the method of genome-wide linkage study (GWLS) was the one available and widely used. The GWLS method is used in families with several sick siblings or in extended pedigrees. It is aimed at detecting in patients blocks of molecular markers that are passed from parents to sick descendants but not to healthy ones. The method allows to localize the gene on the area of 1-10Mb. These extensive areas of chromosomes usually include hundreds of genes; thus, the search for the causative gene in linked locus is a difficult and often impossible task.

Today, a more advanced and widely used method is the method of Genome Wide Association Studies (GWAS). This method was a breakthrough in genetic studies of MDs. It is based on using the HapMap program in conjunction with the technique of high-resolution biochips. As a result of the HapMap Project, the distribution of thousands of polymorphic sites - single-nucleotide substitutions (SNP) was revealed in the human genome and haplotype maps were created - maps of stable combinations of SNP variation within a single-stranded (haploid) DNA sequence [13]. Another important technical achievement was the hybridization of high-density DNA biochips that enabled simultaneous genotyping of thousands of SNP sites in one DNA sample. With knowledge of the exact position of each SNP on the physical map of the haploid genome, it has become not only possible to identify a candidate gene, but also to identify all SNPs associated with MDs [24, 25].

The basis of the GWAS method is in the scanning of hundreds of thousands of markers located on all human chromosomes. Thanks to the haplotypes maps obtained in the HapMap project, modern chip design includes the maximum number of key SNP (tag SNPs) and permits to estimate the frequency of both individual markers and haplotypes for the entire length of the DNA molecule. For example, the widely used chip firm Illumina (www.illumina.com) comprising 310,000 tag SNPs (Illumina Hap310K) enables to estimate the frequency of 81% of frequent polymorphisms in the European population. The next development by the same company comprises 550,000 SNP (Illumina Hap550K) and covers more than 90% of frequent polymorphisms [24].

Genome-wide associations screening is conducted on a large number of patients and controls (more than 1,500 - 2,000 people), which ensures highly reliable (p < 0.000005) results and includes several stages. At first, hundreds of associations are identified, most of which appear to be false-positive after hundreds of thousands of independent tests. In the next step, associations in an independent cohort of patients and controls are analysed by the same method. Only the results confirmed in a replication cohort are considered to be reliably positive. At present, the scanning of about 300 different associations of MDs has been carried out by using GWAS. The results of these studies are summarized on the website of the National Institute of Health (USA) - http://www.genome.gov/GWAstudies/index.cfm≤ # 1. The data include the results of GWAS obtained with a reliability of p < 1 · 10^-5^ and containing not less than 100,000 SNP. They are regularly updated following each publication of new data [25].

**Fig. 5.**
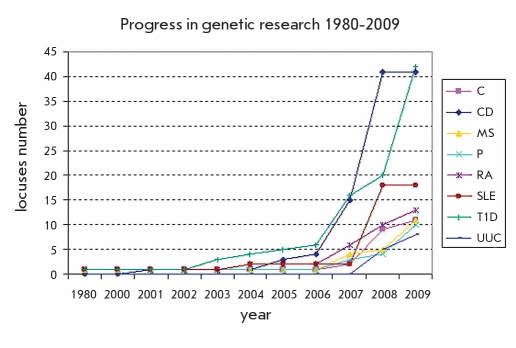
Progress in searching for genes of MD of immune system. C - celiac disease, CD - Crohn’s disease, MS - multiple sclerosis, P - psoriasis, RA - rheumatoid arthritis, SLE - systemic lupus erythematosus, T1D - type 1 diabetes, UUC - unspecific ulcerative colitis.

GWAS in complex diseases is very popular and has been successfully used in the past few years. Data obtained for genes associated with certain diseases of the immune system are presented in Fig. 5.

Thus, the GWAS method is surely becoming the principal method for the search for candidate genes of all MDs. Unfortunately, this revolutionary technology, to my knowledge, so far remains hardly available in Russia. Given the significant populational differences in genetic polymorphism, the implementation of GWAS technology for the identification of candidate genes of MDs in this country is of utmost necessity.

## 5. GENETIC PASS

Many diagnostic centers in Russia currently widely use molecular methods for the diagnosis of genetic diseases and detection of heterozygous carriage of pathological mutations in families at high risk for the preliminary diagnosis of diseases with a late manifestation and for personal identification (genomic fingerprinting). Gradually, genetic testing for predictive medicine is gaining in strength. Obviously, data on both the genome of individuals and entire families and, gradually, individual and family DNA databases are being created. Such an individual DNA database is a "genetic passport (pass)."

Thus, a genetic passport is an individual DNA database reflecting the unique genetic characteristics of an individual, his or her predisposition to some hereditary, multifactorial and other diseases [4, 5, 6, 37].

The information contained in this really unique document should help to avoid the difficult situations in one’s life associated with ignorance of the individual characteristics of one’s genome; ie, the specific characteristics of one’s heredity. These data allow us to more fully fulfill our genetic ability and are of undeniable value for progeny.

The widespread introduction of molecular diagnostics in modern medicine has already made real the idea of a genetic passport. It is already a de facto reality, and the number of GT is increasing rapidly. However, launching the establishment - and especially the practical use - of genetic passports can only be allowed with fairly stringent regulatory requirements. The latter include the following:

a well studied genetic network for each MD;

2. reliable clinical and population data confirming the contribution of relevant marker genes in the pathogenesis of MD;

3. representative data for the population of a particular region or the ethnic group demonstrating the association of the tested marker genes with the MD;

4. a well-balanced (adequate) interpretation of GT results in hereditary predisposition;

5. cogent recommendations based on the results of GT (according to the genetic passport);

6. monitoring of the remote results of the patient’s condition after GT and the prescription of recommendations by clinical geneticists; and

7. privacy, accessibility, and legal protection for patients.

**Fig. 6.**
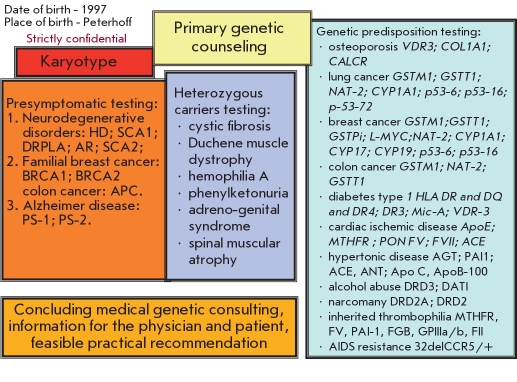
A version of the "genetic passport" [5,6]

The full version of a genetic map should include not only a study of predisposition genes, but also asymptomatic carrying of mutations of genes of the most common hereditary diseases (hemophilia, cystic fibrosis, phenylketonuria, etc.). Currently, the existing molecular diagnostic laboratories in Russia, including those located in St. Petersburg, allow for a fairly complete set of essential genetic tests. One of the first variants of a genetic passport was proposed by us in 1997 (Fig. 6)

At present, practical applications are found for only some components of a genetic passport (heterozygous carrier testing, genomic fingerprinting, and karyotyping). Rarely, and only in high-risk families, is hereditary predisposition to bronchial asthma, diabetes, or osteoporosis tested.

**Fig. 7.**
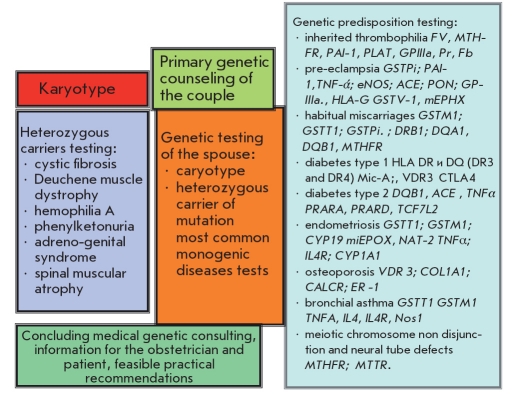
A version of the "genetic card of reproductive health" [26]

The genetic map of reproductive health appears to be more advanced as regards its clinical implementation. Such a map was obtained as a result of many years of comprehensive studies of the reproductive function of women conducted at the Ott’s Institute of Obstetrics and Gynecology RAMS (St. Petersburg) [26]. The map is recommended for use in family planning centers, as well as in antenatal and other centers of the institute. It is widely used in outpatient counseling by clinical geneticists and obstetricians in our institute. In addition to karyotype analysis and testing for a carrier of mutations of severe hereditary diseases for spouses who are planning to have a child, the screening of women for an array of genetic diseases complicating pregnancy, fetal development, childbirth and the postpartum period (pre-eclampsia, habitual miscarriage, varicosity, feto-placental failure) has an important prognostic significance (Fig. 7). For gynecologists and endocrinologists, testing of genetic susceptibility to endometriosis, adenomyosis, and postmenopausal osteoporosis is of great interest.

Particular attention is focused on the testing of hereditary forms of thrombophilia, for diagnostics of which a special microbiochip called "Fibrochip" was developed [27]. The clinical trials of the genetic map of reproductive health (GMRH) conducted at the Ott’s Institute of Obstetrics and Gynecology, RAMS, focused primarily on individual disorders, such as endometriosis (prognosis and choice of optimal treatment tactics), inherited thrombophilia factors, miscarriage and placental insufficiency, and gestosis (prediction and prevention). The accumulated information on the genetic markers of obstetric pathology that results from prospective testing is a good reason for a wider implementation of such a card (GMRH) in clinical practice.

**Fig. 8.**
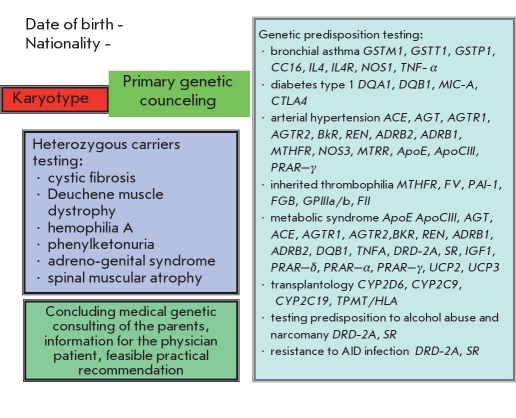
A version of the "genetic card of a child’s health" [37].

Based on WHO recommendations, genetic testing should be carried out with the voluntary, informed consent of the patient; ie, after he or she reaches adulthood. Formally, this means that important genetic information could become available relatively late, when its benefits to the subject and his close relatives have largely been lost. However, taking into account the importance of these data for children’s health, the harmonious development of their personality, rational nutrition, effective education, athletic training, optimal proffessional guidance, and the opportunity to prevent the development of several diseases with a late manifestation, introducing genetic passports at an early age would seem to make sense today. A possible genetic map of a child based on the results of studies of his gene associations is shown in Figure 8.

It cannot be excluded that as the ethical and social problems associated with studies of the human genome are solved genetic testing in an earlier than currently recommended age will become common. In any case, in families with a high risk of diabetes type-1, asthma, sudden death due to cardiac conduction and rhythm disorders, metabolic syndrome and obesity, as well as several other nosology, preventive genetic testing at an early age seems reasonable. [37]. Obviously, the testing should only be conducted with parental consent, with recommendations from a pediatrician, and after consultation of the family by a clinical geneticist competent in predictive medicine.

Likewise, information about gene markers, the testing of allelic variants of which allows to evaluate the suitability of a teenager to a particular sport, accumulates rapidly. Currently, information is available about nearly 150 different genes that control human physical development. That is important for proper fitness and for the selection of potentially promising athletes. The obtained results allowed to start building an individual version of the genetic map of the athlete, which includes the testing of certain genes that determine human physical characteristics.

Despite certain limitations of legal, moral and ethical character, a lack of information on the gene networks of various metabolic processes and multifactorial diseases, the lack of reliable statistical information and the imperfection in the clinical interpretation of the results of genetic testing, the creation of a functioning genetic passport of some type for citizens should be welcomed. Such a medical document can be of substantial assistance during the examination of the health status of an individual, as well as in assessing the potential risk of developing a particular disease by members of a family at high risk of developing an MD [37].

Thus, despite the obvious imperfections of modern predictive medicine, genetic screening of families at high risk for some serious MDs, as well as professional athletes and people exposed to extreme work conditions and individuals simply interested in information about their genome, seems quite real. Obviously, a genetic map of reproductive health is also of great practical significance.

## 6. THE MAIN PROBLEMS RELATED TO GENETIC TESTING OF MD

Testing for gene associations, i.e. the search for candidate genes coupled with a variety of MDs, is already widespread throughout the world, including at leading laboratories and genetic centers in Russia. According to international reports, thousands of polymorphic sites are tested daily to determine associations with diseases [28]. About 100 major candidate genes, each of which contains several polymorphic sites that affect the function of the gene and its products, have already been identified or are under study for common MDs.

The GWAS method in searching for marker genes of MDs has become more widely used in recent years [29]. Hundreds of candidate genes and anonymous polymorphisms coupled with the most common MDs have already been identified using this method [29]. Comparison of the characteristics of the distribution of allelic variants in patients and healthy people has already led to the definition of the "genomic profile" of a MD that corresponds to the SNP-alleles distribution in the genome typical of a particular disease. Thus, identification of marker genes in itself appears not to be important. The results are written using the numbers of tagSNP that have shown reliable linkage with the MD. For example, 22 variants of the genomic profiles of risk of prostate cancer were identified in a large prospective study of the Icelandic population. The highest linkage (OR = 1.23; P = 6.7 · 10^-12^) was found for SNP rs11228565 of locus 11q13. The presence of four variants of the risk SNP rs10934853 (3q21.3), rs16902094, and rs445114 (8q24.21) rs8102476 (19q13.2) increased the probability of prostate cancer in carriers by 2.5 times compared with population SNP variants [30]. Commercial genetic tests, including "individual genomic profiles," have emerged and are been actively promoted by various diagnostic centers in America (Celera, Myriad genetics, Decode, Navigenics, 23andme) and Western Europe (Sciona, Gendia) [31].Yet the associations of numerous marker genes and polymorphic loci with a particular disease remain unproven, and more than 5% of already identified associations turn out to be random [28]. In the opinion of many experts in molecular medicine and medical genetics, a quite alarming situation is developing as commercialization and business become the primary motive, instead of science.

The main problems that have appeared in the implementation of GT results of hereditary predisposition to clinical practice are as follows:

the completeness of identification of all candidate genes of the gene regulatory network (FGM) of MD;

proofs of the reliability of their association with MDs;

medical evaluation of GT results; and

the clinical significance of predictive GT.

## 6.1. THE COMPLETENESS OF THE IDENTIFICATION OF CANDIDATE GENES FGM FOR A PARTICULAR MD

The method of analysis of association (see Section 3) at best permits to reveal the most obvious, in terms of the pathogenesis of the disease, candidates genes and conduct a comparative analysis of allele frequencies in cohorts of patients and population controls.

The method of genome-wide linkage analysis allows for a more detailed identification of loci coupled with a specific MD, but the rather extended size of these loci often highly complicates the exact identification of important candidate genes in it.

Today, hope in solving this problem rests on the GWAS method. Using high-density biochips (over 500,000 SNP) allows to overlap the full genome with high reliability. Thus, information on all the SNP linked with MDs can be obtained. However, many such associations appear to be random in fact and can be discriminated only by repeated GWAS analysis conducted on other cohorts of patients with the same MD and other groups of population control. In addition, the association in one or another DNA site does not always coincide with the presence of the corresponding candidate gene in it and does not necessarily mean the identification of the mutations responsible for the predisposition to the MD. In most cases, the identified SNP sites are located in intergenic DNA and, in fact, can be considered only as a molecular marker linked with one or several neighboring genes. However, at the moment the GWAS method, when complemented by the sequencing of linked loci and the analysis of the expression of its constituent genes, is considered as the most effective method for identifying candidate genes comprising FGM of a particular MD. However, the identification of new marker genes associated with the MD is often paired with the revision of the value of the initial individual risk. That was clearly illustrated in the genomic scanning for predisposition to type 2 diabetes [32].

The possibility of testing for hereditary predisposition not only on the basis of the allelic variants of predisposition genes, but also for the genomic profile on the basis of tagSNP [30] significantly bolters the case for predictive medicine.

## 6.2. THE RELIABILITY OF THE ASSOCIATION OF CANDIDATE GENES WITH A SPECIFIC MD

Currently, there are already about 1,024 clinical genetic tests of MDs and there are more than 300 genetic tests that are in the pre-clinical and clinical trials stages [33]. For many MDs, some "major" genes have already been identified. Their involvement in a particular pathology has been confirmed by research on representative groups of patients in many laboratories. Such MDs include, for example, Alzheimer’s disease (AROE4), type-2 diabetes (PPARG, TCF7L2, KCNJ11), senile macular degeneration of the retina (CFH), systemic lupus erythematosus (JRF5), prostate cancer (region JF1H), type-1 diabetes (IL2RA, CD25, PTPN22), autoimmune thyroiditis (CTLA4), hirschsprung disease (RET), Crohn’s disease (NOD2, CARD15), and rheumatoid arthritis (PTPN22) [8, 34]. Nevertheless, the attitude toward GT remains quite skeptical, largely because of the absence of definite statistical evidence of the reliability of GT results.

One important reason for such a discrepancy is the relatively small sample groups of sick and healthy patients. At best, they are limited to hundreds of subjects, whereas a comparative genetic analysis of several thousands of healthy and sick patients is required to obtain statistically reliable data [28].

Populational differences in allelic frequencies for the same candidate gene could be another reason for the variability of gene associations. Non-randomly identified allelic differences demonstrating the significance of a particular association are often averaged and become statistically insignificant when combined with data from different works carried out on groups of patients, and controls of other populations.

The need for large sample groups is dictated by the relatively low rate of relative risk, OR, which shows how much more common is the studied disease in individuals to study the disease with a specific allele or set of different alleles of the marker genes, compared with individuals with other alleles of the same candidate genes [35]. Typically, for known gene markers associated with an MD, the value of OR does not exceed 1.5 and is usually within 1.16-1.2. Thus, the combination of several unfavorable alleles of candidate PTPN22 in diabetes and systemic lupus erythematosus and the NOD2 gene in Crohn’s disease increases the relative risk of the disease (OR) 2-3 times. For most other associations, OR ranges from 1.1 to 1.5. [22]. Therefore, the real contribution of each marker gene in the development of the MD is relatively small. Therefore, for definite proof of association extensive genetic studies are required. It is believed that to prove a 90% probability of any association between a gene and disease with an OR equal to 1.25, the study requires 5,000 patients and a control group of not less than 5,000 people. This is assumed to increase the level of significance (p <0.00005 instead of the usual p <0.05) 10,000 times [28]. The appearance of the GWAS method has radically changed the situation as relates to the problem of predictive validity of GT. Thus, in assessing the risk of myocardial infarction by GT of 85 SNP in corresponding candidate genes, the reliability of associations has increased to p <0.0000001 [36].

Another important factor that proves the strength of identified association is its replicativeness; ie, the reproducibility of GT in the works of other researchers.

The reasons for the variability of genetic polymorphism are very diverse.

- Genetic stratification of the studied population (the presence of subpopulations with different initial frequencies of the analyzed alleles in it) [28];

- Allele frequencies in different populations may vary, and their contribution to the pathogenesis of a specific MD could be different;

- Pathogenetic differences in a MD could be due to the peculiarities of the action of various external factors in different geographical conditions;

- The inaccuracy of the clinical diagnosis could lead to errors in the formation of clinical sample groups;

- Alleles associated with the same MD in different populations could be different.

There are several other factors that substantially hinder the correct evaluation of the observed genotype-phenotype association, even if it is quite statistically significant:

- The identified association cannot relate to an identified gene or SNP-marker but to a gene or locus (alleles) that is closely linked with a yet unknown locus or allele, whose product is involved in the pathogenesis of MD;

- The identified association could, in fact, be not related to the candidate gene in itself but to another gene product which functionally compensates for the effect of the mutant gene (epistatic relationship of genes);

- Not only gene--gene interaction remains very poorly understood, but also the interaction of candidate genes with environmental factors;

- The pathogenesis of any MD can often be the result of disorder in the function of not just one, but different gene networks;

- Along with the typical polygenic forms of a MD, there are also common forms of monogenic (osteoporosis, Parkinson’s disease, Alzheimer’s disease, various cancers) MDs[35].

Obviously, all these factors significantly complecate the correct identification of marker genes and the assessment of their objective contribution to the pathogenesis of the disease.

Recognizing all these limitations and the very real difficulties in the identification of the marker genes of MDs, it is important to pay attention to the following circumstances:

- All MD genotype--phenotype relationships always have a stochastic nature and are not strictly determined; ie, accuracy in the DNA diagnosis of MDs, in contrast to monogenic diseases, never approaches 100%.

- Groups of sick and healthy patients will include thousands of people in an analysis using the GWAS method, which ensures a high reliability of the results.

- The overwhelming majority of marker genes and loci identified with GWAS usually includes associations previously established by other methods.

These statements need further clarification and verification, but they already provide a basis for more extensive preclinical and clinical trials of the already identified candidate genes of common MD.

At the current stage in development of predictive medicine in Russia, it seems rational to compare the known candidate genes of MDs identified in the works of local researchers with the corresponding panels of candidate genes identified using GWAS (1), to test the allele frequencies of the main new candidate genes on already existing samples of patient-healthy DNA-banks (2), and to create of a new panel of genes of MDs based on the data obtained (3).

## 6.3. EVALUATION OF THE RESULTS OF PREDICTIVE GT

The evaluation of genetic testing results should be conducted considering already existing knowledge about the gene networks of specific MDs, the population, gender, and age characteristics of the frequency of polymorphic alleles of the studied genes [37]. Considering the probabilistic nature of forecasts based on the genetic testing of hereditary predisposition to MDs, some assistance in assessing the risk of a hereditary predisposition can already provide a fairly simple method of score evaluation, which is used in some western countries (Harvard School of Public Health) and is already used in some Russian centers conducting genetic testing [38, 39, 40]. The essence of the method is as follows: each genotype variant is estimated on conditional points depending on whether the alleles identified are protective or predispose to the development of pathology. To this end, alleles with altered functional activity of the gene are assigned 1 point, while normal (frequent), wild-type alleles are assigned 0 point. Then, the points corresponding to genotype for every tested candidate gene are recorded, summed up, and divided by the number of tested genes. So, the risk of developing the disease could be conditionally evaluated as medium, low, or high. In the presence of pathogenetically complex MDs, including several different metabolic chains, the calculation is conducted for each gene network separately and the estimates obtained are added up. Some variants of score evaluations, in addition to the calculation of points corresponding to genotypes, also include conditional points of various exogenous factors (harmful effects, habit, medication intake, etc.), anthropometric values, as well as physical activity, sex, weight [26].

Information on the basis of GT results is prepared by a clinical geneticist, together with the specialist who carried out the molecular analysis, and is passed along to the physician and patient. Comparison of this conclusion with the results of clinical, laboratory, and instrumental investigations permits to assess the risk of developing a MD more objectively and to offer the most effective program of prevention and treatment.

The answer may be more objective if it is possible to compare the results with data on the genetic testing of a close relative who has a given MD. But in any case the answer would be essentially probabilistic in nature. A detailed method of scoring the results of GT is given in our user guide devoted to the genetic map of reproductive health [26].

Special computer programs could significantly assist in the correct interpretation of GT results. Such programs are available and are used to compare the genomic profiles of patients with MDs, the control group of patients, which allow us to estimate the risk of hereditary predisposition of a particular person to a MD [29]. A map is created that facilitates the understanding of the results of GT by the medical staff. It helps to find genetic variations corresponding to certain diseases and to monitor their transmission by inheritance. It is believed that such a map will help to reduce the cost of searching for genes of predisposition to a particular MD, as well as to make real development of individual treatment possible [41]. There is no doubt that the establishment of such a computer program to evaluate GT results in Russia would also greatly accelerate the introduction of predictive medicine in clinical practice.

According to foreign reports, GT for assessing genetic susceptibility to cardiovascular diseases, venous thrombosis, hyperlipidemia, and atherosclerosis has already been developed and is widely used [41]. It is important to emphasize that these studies have yet to acquire the status of tests recommended for clinical use, but they are already being used by clinicians. More than 1,000 predictive GT, many of which are at the pre-clinical and clinical trials stages are in the same state. The objects of GT are such genes as APOE4, homozygosity for which increases the risk of Alzheimer’s disease 14-fold; gene Filaggrin, mutation in which R501X or 2282del14 increases the risk of atopic eczema and severe asthma 4-fold; the unfavourable alleles of CDKN22a2b (64% increase of risk of infarction by 64%); and 4a/4b alleles of IN, the presence of which doubles the risk of type 2 diabetes. However, the clinical significance of these genetic tests remains unclear; their usefulness to physicians and patients needs more rigorous proof [36].

The first predictive genetic test to calculate the individual dose of the anticoagulant warfarin successfully passed certification and received the formal approval of the U.S. Food and Drug Administration on August 16, 2007. The test includes testing of the genes CYP2C9 and VKORC1, taking into account the age, sex and weight of the patient.

In 2008, the EuroGentest Commission in Europe developed guidelines for GT standardization and prepared the necessary documentation for certification of those genetic tests that, on the basis of the results of clinical trials, can be referred to the category of GTB recommended for clinical use [Nippert et al., 2008].

## 6.4. RECOMMENDATIONS ON THE RESULTS OF GENETIC TESTING

GT may offer real benefits only if it is accompanied by full consultation with a qualified clinical geneticist, with the provision of relevant recommendations to the physician and patient. GT may have practical value if the following conditions are fulfilled: (1) the results of GT are based on the analysis of genes whose association with the corresponding disease is shown in the population of the particular region; (2) the subject is a family member at high risk, where there is already a patient with this pathology; and (3) the GT data are subjected to adequate statistical analysis. Effective use of such information is largely determined by the level of genetic knowledge of physicians, their ability to apply the data to the diagnosis, prevention and treatment of diseases, as well as the willingness of the patient to follow the recommendations of physicians based on the results of genetic testing. [39]. But even under these conditions, GT results concerning hereditary predisposition should be interpreted very cautiously. When possible, GT should be complemented with an appropriate biochemical analysis allowing to evaluate the functional activity of the studied genes. It should be required that more objective information be obtained by testing the genes controlling only a single metabolic process; ie, the genes belonging to the same gene network. Thus, already today on the basis of genetic testing the functional conditions of the following systems can be assessed objectively: the detoxification system, blood coagulation, lipid or carbohydrate metabolism, the renin-angiotensin system, and others. Assessing the outcome and prognosis of hereditary predisposition to MDs caused by damage to several gene networks is much more difficult.

The main difficulties in the widespread implementation of predictive medicine in clinical practice are related to the lack of objective data to prove the usefulness of preliminary (no symptom) testing of hereditary predisposition to MDs for the patient.

According to the Gene Dossier recently developed by the United Kingdom Genetic Testing Network (www.ukgtn.nhs.uk), certification of each new GT should include (1) information on the analytical precision of the used molecular genetic methods; (2) the clinical reliability of the GT, i.e. its ability to diagnose or predict the presence or absence of a certain phenotype; (3) the clinical usefulness of GT, its compliance with ethical, legal and social norms, which means that GT should be aimed at specific populations and serve to solve a particular problem (www.labtestonline.org.ru).

The British fund Wellcome Trust, which initially funded the Human Genome Project program, in 2008 began supporting a project aimed at improving and strengthening the evidence base of genetic testing, as well as the development of a handbook for the coordination and integration of GT into clinical practice with sufficient explanations of their usefulness to physicians and patients. At the same time, the estimation of the clinical usefulness of GT is equivalent to phase III of clinical trials, but it remains unclear whether GT should be paid for by governments or the firms working on and advertising GT [43].

The ultimate goal of the project is to move predictive medicine from the field of research of genetic polymorphism features and identification of genes - markers of MDs to the level of evidence-based medicine.

The results of every GT should include not only information about the features of the allelic variants of the genes of a metabolic chain, but also recommendations for the patient and physician [44]. A "Genetic" reorientation of the health care industry is already happening in the developed countries of Western Europe and America. In the near future, it will reach Russia. A significant improvement in genetic knowledge, especially in the field of predictive medicine, for doctors in all specialties is very important for the effective implementation of the achievements of medical genetics and genomics in the Russian health care system.

## CONCLUSION

Thanks to impressive advances in genomics, the emergence of new, highly effective methods of molecular analysis, the search for marker genes associated with MDs has rapidly developed. As a result, (1) thousands of new marker genes, the allelic variants of which predispose to the development of pathological processes, have been identified; (2) the genetic panels of the most frequent chronic diseases has been established; and (3) marker genes defining the severity of the disease and predisposition to some complications are being identified.

The unprecedented scale of the genotyping of representatives of different races, nationalities, and ethnic groups has required hard joint work from clinicians and molecular biologists. Their work has led to numerous DNA databanks containing information on all known mutations and DNA alterations associated with chronic diseases (4).

Extensive information on the genotyping of frequent chronic diseases among inhabitants of Russia has also been collected at many Russian scientific centers such as the Institute of Medical Genetics of RAMS (Tomsk); Medical Genetics Research Center (Moscow); Institute of Biochemistry and Genetics, Academy of Sciences (Ufa); N.I. Vavilov Institute of General Genetics, RAS (Moscow); Institute of Molecular Genetics, Academy of Sciences (Moscow); and the Institute of Molecular Medicine, RAMS, etc.). Only in our laboratory at the Ott’s Institute of Obstetrics and Gynecology, RAMS (St. Petersburg), has the the frequency of allelic variants of 80 marker genes in 5,000 patients with different parts of MDs and the same number of people from the control group been studied.

As an individual DNA database, the genetic passport (GP) already exists and is gradually becoming more complex with the identification of new genetic markers and the increase in the number of gene networks and panels of predisposotion genes determining hereditary susceptibility to MDs. If at its early stages GP was a fairly simple map including test results for approximately 100 marker genes corresponding to gene panels of 15-20 frequent chronic MDs, since the emergence of the GWAS technology the number of candidate genes has grown rapidly. Its characteristic genetic profile corresponding to the distribution in the genome of more than 30-500, 00 SNP in the SNP map could be determined for each disease with the help of this technology. Comparison of the genetic profiles of sick and healthy patients with that of a particular subject provides a high degree of reliability in testing inherited predisposition to the relevant disease.

It is obvious that GT results have to be treated with great caution. The clinical usefulness of such testing, even with GWAS technology, still needs to be proved. Of particular concern is the lack of information on how and what environmental factors provoke the onset of MDs in a particular person. To this end, the scientific community has already been tasked with quantifying the genetic and exogenous risk factors and their combinations in the pathogenesis of MDs [45]. Despite the serious complexity, the implementation of predictive medicine in clinical practice is scientifically founded and strategically unavoidable.

In conclusion, let me note that the emergence of new, highly efficient methods of DNA sequencing has made it possible to sequence the full genome of an individual. Massive parallel sequencing is particularly promising in this respect [46]. Recently, it was reported that each American can now get his or her whole genome for $ 50,000; ie, 20 times less than in 2007. It should be noted, however, that the completely sequenced genome of an individual is unlikely to replace the genetic passport in the near future. A genetic passport is much more convenient and practical in everyday use for a clinical geneticist and for a physician using the GT data. The full genome sequence will certainly be important for a more in-depth analysis of the unique features of the individual genome; ie, it can play the role of a universal genetic handbook for everyone, while GP will provide information on the status of the predisposition genes of several common MDs. It is assumed that within the next 2-3 years, everyone will be able to obtain a complete set of his genome for $1,000, and that the cost of a genetic passport with the commentary of a specialist will cost about $300. Hence, in addition to conventional major medical tests and an anthropometric survey, the personal medical card of each person will also include the results of GT, the number of which will invariably increase. However, according to the opinion of academician V.P. Pusyrev, GT will not replace but will only supplement the results of other laboratory studies [20].

Anticipating such further development, a lot of work on the integration of genomics research in the national (public) health policy and practice has already been carried out in the countries of Western Europe and North America. Knowledge of the genome seguence has to be integrated into the health doctrine of each country, with emphasis on predictive medicine. To do this, however, there is still the need to figure out what clinical value "high genetic sensitivity" represents and how to quantify genetic and exogenous risks in accordance with the principles of evidence-based medicine [45].

The advances of genomics in society and in medicine can be accelerated, but it cannot be halted. This fully applies to predictive medicine. The introduction of the technology of genome-wide screening for the identification of candidate genes of MDs, the comparison of the individual profiles of the allelic variants of candidate genes of a subject with those of sick patients with a particular MD and of obviously healthy people supported by the outcomes of prospective genetic testing will open a wide avenue to a new and promising era of predictive medicine for humanity. The main task of modern genomics is to evaluate the significance of the results of GT to determine the conditions of their implementation in medical practice. Possible solutions to this problem in Russia include the following: (1) a comparison of the obtained GT results of MDs of local populations with international data of their genome-wide screening, (2) the establishment of representative (at least 1,000 samples) DNA banks for each MD, (3) testing of new candidate genes on already existing domestic DNA collections, and (4) organizing centers for the implementation of genome-wide association screening GWAS. 

## Acknowledgements

The authors express their deep appreciation to the researcher of the Department of Biomedical Genetics, University Medical Center of Utrecht (Netherlands) A.P. Zhernakova for her valuable advice on the manuscript and the possibility to use her data concerning GWAS of a number of autoimmune diseases.
